# Unmet Needs and Current Challenges of Rheumatoid Arthritis: Difficult-to-Treat Rheumatoid Arthritis and Late-Onset Rheumatoid Arthritis

**DOI:** 10.3390/jcm13247594

**Published:** 2024-12-13

**Authors:** Satoshi Takanashi, Yuko Kaneko

**Affiliations:** Division of Rheumatology, Department of Internal Medicine, Keio University School of Medicine, 35 Shinanomachi, Shinjuku-ku, Tokyo 160-8582, Japan

**Keywords:** rheumatoid arthritis, difficult-to-treat rheumatoid arthritis (D2T RA), JAK inhibitors, late-onset rheumatoid arthritis, elderly, aging

## Abstract

Despite remarkable advances in the management of RA, there are still unmet needs that rheumatologists need to address. In this review, we focused on difficult-to-treat RA (D2T RA) and late-onset RA (LORA), and summarized their characteristics and management. The prevalence of D2T RA is reported to be 6–28% and many factors have been identified as risk factors for D2T RA, including female sex, long disease duration, seropositivity for rheumatoid factor and anti-cyclic citrullinated peptide antibody and their high titer, baseline high disease activity, and comorbidities. D2T RA is broadly divided into inflammatory and non-inflammatory conditions, and clinical features differ according to background. A proportion of D2T RA can be managed with treatment modification, mainly with interleukin-6 receptor inhibitors or Janus kinase inhibitors, but some D2T RA patients have a poor prognosis; thus, the implementation of precision medicine by stratifying patients according to disease status is needed. In the aging society, the epidemiology of RA is changing and the prevalence of LORA is increasing worldwide. LORA has distinct clinical features compared with young-onset RA, such as acute onset, low seropositivity, and high inflammation. The pathogenesis of LORA remains to be elucidated, but proinflammatory cytokines, including interleukin-6, have been reported to be significantly elevated. LORA has several management concerns other than RA itself, such as geriatric syndrome and multimorbidity. The treat-to-target strategy is effective for LORA, but the evidence is still lacking; thus, it is important to accumulate clinical and related basic data to establish the optimal treatment strategy for LORA.

## 1. Introduction

The landscape of rheumatoid arthritis (RA) has changed dramatically in recent decades. The development of the 2010 American College of Rheumatology (ACR)/European Alliance of Associations for Rheumatology (EULAR) classification criteria [1], which allows for the early diagnosis of RA; the treat-to-target (T2T) strategy [2]; novel treatments, such as biologic disease-modifying anti-rheumatic drugs (bDMARDs) and Janus kinase inhibitors (JAKis) (Table 1) [3,4]; and the widespread use of evidence-based EULAR recommendations for RA [5] have contributed to a paradigm shift in the management of RA. As a result, the achievement rate of the treatment goal has improved remarkably [6], and the life expectancy of patients with RA is increasing [7]. Furthermore, research is underway to establish disease prevention and chronicity techniques in preclinical RA [8,9]. Despite the remarkable progress in the management of RA, there are several unmet needs that rheumatologists need to address in the future, such as difficult-to-treat RA (D2T RA), in which treatment goals are not achieved for multifactorial reasons [10,11], as well as late-onset RA (LORA) in the super-aging society [12,13]. In this review, we focus on these unresolved issues in the field of RA and discuss how they can be addressed in the future.

## 2. Difficult-to-Treat Rheumatoid Arthritis (D2T RA)

### 2.1. Concept and Definition of Difficult-to-Treat RA (D2T RA)

There is a proportion of patients who remain symptomatic after several cycles of treatment based on EULAR recommendation and who fail to achieve the treatment goal; this population has been recognized as a significant clinical problem [10]. They have been described as refractory, treatment-resistant, severe, complex, or difficult-to-treat, but there has been a lack of consistent terminology to define this population. To address the great unmet need, the EULAR Task Force conducted an international survey among rheumatologists to provide terminology and a definition of D2T RA as an essential first step. This international survey on the disease characteristics of D2T RA was conducted among 410 rheumatologists in 2018. In the survey, 50% of them answered ‘disease activity score assessing 28 joints using erythrocyte sedimentation rate (DAS28-ESR) >3.2 or presence of signs suggestive of active inflammatory disease activity with a DAS28-ESR ≤ 3.2’ as being characteristics of D2T RA [11]. Also, 62% answered ‘≥1 or 2 conventional synthetic (cs) DMARDs and ≥2 b DMARDs or targeted synthetic (ts) DMARDs with different modes of action’ as being the minimum number of inadequately effective DMARDs that should have been applied. Based on the results, EULAR proposed a definition of D2T RA in 2021 [14] (Table 2). Briefly, all three criteria need to be present in D2T RA, as follows: first, a treatment failure of 2 ≥ bDMARDs with a different mechanism of action after failing csDMARDs treatment; second, signs suggestive of active or progressive disease, such as moderate or high disease activity according to the validated composite measures, inability to taper glucocorticoid treatment, rapid radiographic progression, and symptoms that are causing a reduction in quality of life; third, the perception of the rheumatologist and/or patients that the management of RA is problematic.

### 2.2. Prevalence of Difficult-to-Treat RA

At the time the concept of D2T RA was developed, its prevalence was estimated to be around 3–10% of all RA patients [10]. Since the EULAR definition of D2T RA has been proposed, several real-world studies of D2T RA have been published and the actual prevalence of D2T RA ranges from 5.9 to 27.5% in clinical practice (Table 3) [15,16,17,18,19,20,21,22,23,24,25,26,27,28,29,30,31]. The reasons for the variation in the prevalence of D2T RA may be due to differences in the patient populations included (proportion of early RA, established RA), healthcare systems, type of institute (academic hospital, general hospital, clinic), and access to drugs.

The accumulation of evidence has revealed several concerns about the definition of D2T RA. Firstly, how to deal with older patients with comorbidities. We verified the definition in our institution, where approximately 60% of 1700 patients were treated with b/tsDMARDs, resulting in approximately 85% achieving target (60% in remission and 25% in low disease activity), and revealed that 10% are classified as D2T RA [16]. In the process of this research, we also identified 77 patients (5%) who were non-D2T RA despite being in moderate or high disease activity due to the non-fulfillment of the criterion of the failure of two b/tsDMARDs with different mechanisms of action [32]. This population was older at diagnosis and last visit (62.3 and 77.5 years, respectively), and had more comorbidities, including lung and kidney disease (41.6 and 50.6%, respectively), which made them or their attending physicians reluctant to intensify or change the treatment. These data suggested that we should pay more attention to patients with RA who do not meet the current criteria because of safety concerns.

Secondly, how to deal with the patients who have limited access to bDMARDs or JAKi due to the socioeconomic reasons [16]. Because of the high cost of the drugs, some patients with an inadequate response to methotrexate (MTX) are unable to try bDMARDs or JAKi. In the definition of D2T RA, the following is described in the footnote: “failure of ≥2 b/tsDMARDs unless restricted by access to treatment due to socioeconomic factors”; therefore, these patients are also included as having D2T RA. The definition of D2T RA is a broad concept that includes social factors, so it is necessary to recognize that D2T RA is a heterogeneous condition.

These real-world data highlight the performance and concerns of the EULAR definition in clinical practice.

### 2.3. Clinical Characteristics and Risk Factors for D2T RA

In general, many factors may be complicatedly associated with the condition of D2T RA, such as immunologic mechanism, pharmacogenetics, smoking, immunogenicity of bDMARDs, adverse drug reactions, medication non-adherence, and inappropriate medication use [10]. In addition, factors surrounding the patients such as comorbidities, obesity, and joint damage may affect the signs and symptoms of RA. We have summarized the factors contributing to D2T RA from the real-world data in Table 3 [15,16,17,18,19,20,21,22,23,24,25,26,27,28,29,30,31]. There are many factors including female sex; young onset; long disease duration; treatment delay; seropositivity for rheumatoid factor (RF) and anti-cyclic citrullinated peptide antibody (anti-CCP); multiple comorbidities, especially lung disease, chronic kidney disease, and fibromyalgia; and baseline high disease activity. These data suggested that D2T RA is a highly heterogeneous and multifactorial condition. Interestingly, these factors have been shown to work synergistically to increase the risk of D2T RA. In one study, RF titer, baseline DAS28, and co-existing pulmonary disease were extracted as risk factors of D2T RA, and the combinational score of the three factors was positively correlated with the futuristic development of D2T RA [17]. Another study showed that treatment delay, female gender, and higher disease activity were independent predictors of refractory disease course and that a matrix risk model using these factors efficiently predicts patients’ progression to D2T RA [33].

### 2.4. Subgroup of D2T RA

As D2T RA is a heterogeneous condition, it is important to stratify patients appropriately for optimal treatment (Table 4). At first, patients are usually divided into two groups according to the presence or absence of inflammation [34]. Inflammatory D2T RA is characterized by a lack of response to multiple DMARDs and persistent signs of inflammation. On the other hand, non-inflammatory D2T RA has also been exposed to multiple DMARDs and remains symptomatic, but there is little objective inflammation. In this condition, the effects of accumulated damage and/or secondary osteoarthritis, functional decline, chronic pain syndrome and/or fibromyalgia, as well as central sensitization, also modify the symptoms of RA. In a cross-sectional study comparing the clinical features of inflammatory and non-inflammatory D2T RA based on ultrasound findings, 57% of patients with D2T RA had residual synovitis in one or more joints, while the remaining 40% had no detectable inflammation. These two groups were clinically distinct—the inflammatory group had an increased swollen joint count (5.0), high CRP levels (1.0 mg/dL), and high DAS28-CRP (5.3), while the non-inflammatory group had a higher proportion of obesity (55%) and fibromyalgia (15%) [27]. A previous study suggested that such residual pain in non-inflammatory D2T RA is attributable to persistent central sensitization and the development of maladaptive pain processing [35]. Another study reported that synovial fibroblast lining cells may be involved in dorsal root ganglion axonal growth and cause low inflammatory intractable pain [36]. Further detailed elucidation of the mechanism is required for appropriate treatment.

There are several reports on subgroups of D2T RA from real-world cohort studies. In our study, we divided into three groups based on medical record information and the opinion of attending rheumatologists—the multidrug resistance group, the comorbidity group, and the socioeconomic group [16]. We found that the clinical characteristics differed between the groups. The multidrug resistance group had the highest tender joint count (3.4 joints) and evaluator global assessment score (21.9 mm) of the three groups, despite having tried an average of 3.9 different bDMARDs/JAKis. The patients in the comorbidity group showed the highest rate of admission due to infection (64.7%), the highest score of rheumatic disease comorbidity index (1.9), and the most frequent comorbidities of lung involvement (52.9%). They were also significantly older (75.3 years), had the longest disease duration (18.4 years), and showed the smallest physique.

The other study that conduced cluster analysis using the clinical characteristics of D2T RA showed that there are three subgroups—non-adherence and dissatisfaction group, pain syndromes and obesity group, and true refractory RA [15]. This study showed that co-existing factors not directly related to the pathology of RA, such as obesity, anxiety, depression, fibromyalgia, and treatment non-adherence, were also important in stratifying D2T RA.

Therefore, D2T RA can be stratified according to its clinical features, reflecting the heterogeneity of this state, and a personalized approach will be needed to resolve the D2T RA.

### 2.5. Management and Treatment Strategy of Difficult-to-Treat RA

EULAR has proposed points to consider and an algorithm for the management of D2T RA [37]. This algorithm proposes a multifaceted approach, including not only pharmacological but also non-pharmacological intervention, because the reasons for the difficulties in the management are different for each individual. First, this proposal emphasizes the need to reassess the diagnosis of RA from other diseases. If a patient is suspected of having D2T RA, the possibility of misdiagnosis and/or the presence of a co-existing mimicking disease should be considered. Once the diagnosis of RA has been re-confirmed, a treat-to-target strategy should be implemented in accordance with the EULAR recommendation and also the residual inflammation should be assessed using ultrasound. Regarding the choice of treatment among bDMARDs and JAKis, it is recommended to switch to the other mode of action, and there are also some real-life data to support this strategy [16,38]. Additionally, it was mentioned that the maximum dose acceptable from a safety point of view should be used. In addition to pharmacological treatment, we should focus on non-pharmacological management, including education, exercise, and self-management, to optimize the management of functional disability, pain, and fatigue.

We have reported the long-term follow-up data of patients with D2T RA [39]. Our data showed that patients who resolved D2T RA more frequently underwent treatment changes, suggesting that further treatment modification was important for the management of D2T RA. In this study, 80% of patients who resolved D2T RA were treated with IL-6i or JAKis, and logistic analysis showed that the use of IL-6i was a relevant factor for the resolution of D2T RA. Another study demonstrated that JAKi treatment had the highest CDAI remission rate in patients with D2T RA compared to TNFi, IL-6i, and CTLA4-Ig [18]. A further study revealed that JAKi and RTX showed the highest DAS28-ESR remission rate in patients with D2T RA [24]. A recent study involving 450 patients with D2T RA revealed that IL-6i and JAKi were associated with fewer discontinuations due to ineffectiveness compared to TNFi [31]. These data suggest that IL-6i and JAKi may be the preferred choice for the management of D2T RA. Recently, the overexpression of the type 1 interferon signature has been suggested as one of the key cytokines involved in D2T RA [40,41]. Several data have suggested that JAKis, which can suppress the interferon pathway, and IL-6is show the preferred treatment response in patients with type 1 interferon overexpression [19,42]. However, as the pathophysiology of RA is heterogeneous and the optimal treatment target may differ among individuals, it is necessary to establish an appropriate method to stratify patients with D2T RA based on their immune status using transcriptome, gene expression, cytokines, or clinical data, which can lead to precise treatment to overcome D2T RA.

Evidence for non-pharmacological interventions in D2T RA is limited. It would be difficult to accumulate and assess the data on non-pharmacological interventions in retrospective studies; therefore, prospective non-pharmacological interventional programs should be conducted that could help to optimize the management of D2T RA in terms of pain, fatigue, and functional disability.

### 2.6. Outcome of D2T RA

There are few reports on the outcome of D2T RA. We identified 173 patients with D2T RA [16], followed them for 5 years, and investigated their outcome [39]. During the 5-year follow-up, 23 patients were lost to follow-up. Of the 150 patients with D2T RA, 45% resolved D2T RA, 50% had persistent D2T RA, and 5% deceased. Patients who resolved D2T RA significantly changed their treatment during the five years, and the use of IL-6 receptor inhibitors was associated with preferable outcomes. On the other hand, the relevant factors for poor prognosis in D2T RA were multiple comorbidities and glucocorticoid dose escalation. Multimorbidity was known to be a strong predictor of a poor outcome of RA from the cluster analysis based on the number of comorbidities [43]. Indeed, Watanabe et al. also demonstrated that the use of oral glucocorticoids is associated with a risk of toxic side effects in patients with D2T RA [31]. Glucocorticoid use is known to be associated with numerous side effects, including infection, cardiovascular disease, and osteoporosis [44,45]. These results suggested that a portion of patients with D2T RA can be managed by further treatment modification and that a treatment strategy without glucocorticoids is important to overcome D2T RA.

Another study investigated the long-term functional trend in D2T RA [29]. Trajectory analysis revealed four distinct functional HAQ trends. About 20% of D2T RA patients had a more favorable function, with mHAQ scores of 0.41 that remained stable during follow-up. Approximately 40% of the D2T RA patients showed a gradual improvement (mHAQ from 1.21 to 0.87). On the contrary, two groups with unfavorable functional status were identified, of which about 30% had a gradual worsening of functional status (mHAQ from 0.68 to 1.10) and 10% had stable and significant functional limitations throughout follow-up, with mHAQ scores > 1.5. The presence of mental health- and pain-related conditions or metabolic diseases had a significant contribution to the worsening of the mHAQ, suggesting that a special focus on comorbidities, together with better control of the inflammatory burden, could improve the outcome of patients with D2T RA.

Inadequate disease control of RA is strongly associated with all-cause mortality, cardiovascular disease, cancer, and respiratory disease [46]. Co-existing comorbidities also have a strong impact on the prognosis of D2T RA; it is necessary to manage and try to escape D2T RA by adapting the appropriate treatment and thorough management of comorbidities to improve their outcome.

**Figure 1:**
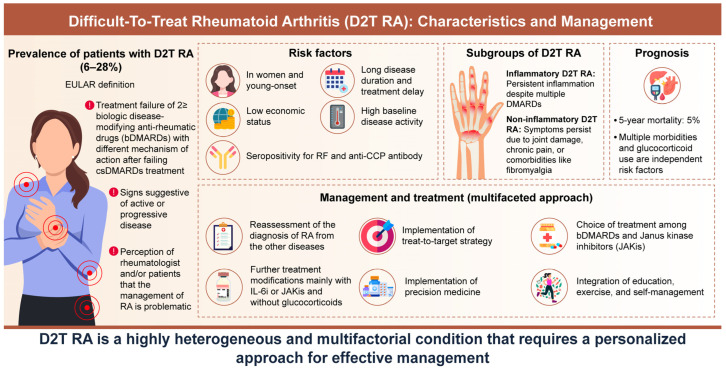
**Clinical characteristics and management of difficult-to-treat rheumatoid arthritis (D2T RA).** The prevalence of D2T RA is 6–28% in the real-world, and female, young onset, long disease duration, treatment delay, high disease activity, low economic status, and seropositivity are risk factors. D2T RA can be divided into two groups with or without residual inflammation. The 5-year mortality is 5%, and comorbidity and glucocorticoid use are risk factors. D2T RA is a highly heterogeneous and multifactorial condition that requires a personalized approach for effective management.

We summarize the clinical characteristics and management of D2T RA in Figure 1.

### 2.7. Future Prospectives for Preventing D2T RA

There have been no clinical trials in D2T RA to date and this should be considered in the future. However, ideally, preventive strategies should be implemented to avoid increasing levels of D2T RA in the future. It is crucial to identify the optimal therapeutic target molecule at an early stage of RA. Currently, it is difficult to predict an individual’s response to targeted therapy, so treatment guidelines are based on a trial-and-error approach, in which a molecular targeted treatment is given and if it does not work, the therapeutic target is changed. However, repeated treatment failures are known to lead to the development of D2T RA, so the implementation of precision medicine by stratifying patients according to disease status and using state-of-the-art methods such as identifying the molecular biology of RA; machine learning will improve treatment response rates. In particular, basic research on synovial fibroblasts and dendritic cell precursors should be targeted to address this condition [36,47].

## 3. Late-Onset Rheumatoid Arthritis (LORA)

### 3.1. Epidemiology and Clinical Characteristics of LORA

The epidemiology of RA is changing and its prevalence has increased in recent decades [48]. In particular, autoantibody-negative, so-called ‘seronegative’, RA is increasing, and the majority of this population suffers from late-onset rheumatoid arthritis (LORA) [49]. In line with this trend, the age at onset of RA has been increasing worldwide, and the onset age in the histogram demonstrated a major peak around the ages of 60 to 70 [50,51]. Given the aging society worldwide, the number of LORA patients is expected to increase in the future [49]. However, there is no consensus on the definition of LORA, with many using 60 or 65 years as a common cut-off [13].

LORA has been reported to differ from young-onset RA (YORA), which is generally defined as an age of onset of RA of less than 60 or 65 years and does not include juvenile idiopathic arthritis, not only in age of onset but also in several clinical features [51,52,53,54,55,56]. Specifically, patients with LORA are known to be typically negative for autoantibodies including anti-CCP and RF, and have a more acute onset, proximal joint involvement, and higher inflammatory markers, making it sometimes difficult to distinguish from polymyalgia rheumatica (Table 5). Multivariate analysis showed that cases with three factors extracted as independently associated with LORA—anti-CCP antibody negativity, acute onset, and high erythrocyte sedimentation rate—were considered typical of LORA, with the number of relevant cases gradually increasing from 65 years of age. When LORA was defined as a patient fulfilling all three factors, a cut-off age of 68–73 years was found using receiver operating curve analysis [51].

LORA has also been reported to be more prone to progressive bone destruction [53,54]. One study reported that the improvement in DAS28-ESR at 12 months was as good in LORA as in YORA, but residual synovial thickening and power Doppler signals assessed using ultrasounds and radiographic progression were significantly worse in LORA [53]. Similarly, in a study comparing the proportion of radiological progression in LORA and YORA that was free of bone erosions at the time of RA diagnosis and achieved remission after a further 1–2 years, bone erosions were significantly more common in LORA [54]. In addition, patients with bone erosions had a significantly higher disease activity at diagnosis. These results suggest that LORA differs from YORA not only in the age of onset but also in the clinical features of RA itself, and that even if disease activity can be controlled, there is a high risk of radiological progression; therefore, early diagnosis and treatment is needed.

### 3.2. Pathogenesis of Late-Onset Rheumatoid Arthritis

Although the clinical features of LORA are distinct from those of YORA, the underlying immune status remains unclear. A study using synovial biopsy revealed that the baseline synovial pathotype, including lympho-myeloid, diffuse-myeloid, and pauci-immune-fibroid, did not differ between LORA and YORA [53]. After 6 months of treatment, there was a trend towards a higher frequency of the pauci-immune-fibroid pathotype in YORA, coupled with a reduction in the frequency of the lympho-myeloid pathotype. In addition to the synovial pathotype, the overall synovitis inflammatory score and macrophage (lining/sublining), T cell, B cell, and plasma cell infiltration did not differ between the two groups at baseline and 6 months. However, YORA patients demonstrated a significant decrease in all synovial inflammatory parameters, whereas LORA presented only mild, albeit significant, decreases in synovitis, sublining macrophage, and T cell scores, with no significant changes in lining macrophages, B cells, and plasma cells. To summarize, although no obvious difference between LORA and YORA was detected via the immunostaining of bulk synovial tissue at baseline, differences in synovial tissue changes after treatment were observed, with a reduced inflammatory cell infiltration in YORA, suggesting that more high-resolution strategies, such as single-cell analysis, may help to elucidate and understand the differences in the pathogenesis of LORA and YORA.

With regard to serum cytokines, significantly higher levels of IL-6 were found in patients with LORA, especially in those with polymyalgia rheumatica-like symptoms, compared to those with YORA [57]. On the other hand, levels of serum tumor necrosis factor-α were significantly lower in patients with LORA than in those with YORA. Studies using synovial fluid have also reported significantly higher levels of IL-6 in LORA compared to YORA. It is known that there is a chronic inflammatory state called ‘inflammageing’, which is associated with increased IL-6 in older people [58]. In addition, cellular stress responses with the release of IL-6 increase with age, contributing to the persistent inflammation seen in older people [59,60]. As the fundamental differences between late-onset and young-onset RA are poorly understood, a multifaceted analysis combining immunophenotyping and cytokine analysis using synovial tissue and peripheral blood with clinical features and treatment response data would contribute to a detailed understanding of the pathogenesis and to the establishment of optimal treatment strategies for LORA.

### 3.3. Management of Late-Onset Rheumatoid Arthritis

In the management of LORA, there are many issues other than RA itself, including geriatric syndromes such as frailty, dementia, physical function, and organ dysfunction, as well as comorbidity, which are complex and intertwined with each other, making it a very difficult condition to address, which has been referred to as the ‘spaghetti model’ [61]. To manage the complex ‘spaghetti model’, the 5Ms approach—Mind, Mobility, Medication, Multicomplexity, and Matters Most—is proposed, which focuses on geriatric care and can help to develop a management plan tailored to the needs of older people.

Regarding the treatment of RA, a consensus statement on LORA has now been published [62], recommending methotrexate as primary treatment, with consideration of molecular-targeted agents if treatment goals cannot be achieved. Achieving a treat-to-target strategy has been shown to be associated with high remission rates without increasing the rate of adverse events in patients with LORA [63]. In this study, the presence of lung lesions and malignancy were independent risk factors for adverse events, while age, methotrexate use, and bDMARDs use were not extracted as relevant factors; therefore, a treatment strategy similar to YORA is desirable, with due consideration for safety. The Glucocorticoid Low-dose in Rheumatoid Arthritis (GLORIA) trial, a recent pragmatic randomized trial, investigated the efficacy and safety of add-on low-dose prednisolone (5 mg/day) in patients aged 65 years with established RA [64]. Add-on low-dose prednisolone therapy shows beneficial long-term effects in older patients with RA, with a trade-off of a 24% increase in adverse events, which mainly consisted of infection. However, this study did not focus on newly onset LORA, and long-term glucocorticoid use (>2 years) is associated with cardiovascular diseases [44], osteoporosis [45], severe infectious adverse events [65], and sarcopenia [66]; thus, we need to be careful about glucocorticoid use, and it is mentioned that the glucocorticoid dose should be kept at a minimum and should be discontinued within 6 months whenever possible in the consensus statement [63].

With regard to geriatric syndromes, the progression of frailty is a strong predictor for subsequent hospitalization and mortality in patients with LORA, and once it has progressed, frailty is difficult to reverse even after an improvement in the disease activity of RA [67]. Comprehensive strategies such as diet, exercise, and vaccination to prevent frailty are considered as important as RA management [68].

Multimorbidity is also an important aspect in the management of LORA; for instance, chronic kidney disease and lung disease may limit the use of methotrexate. An observational study has shown that a group of patients with the highest number of complications had a higher mortality rate when categorized by the number of comorbidities [43]. In addition, a higher number of complications leads to a higher number of drugs administered, and polypharmacy, especially the administration of more than 10 drugs, is associated with difficulties in controlling disease activity in RA and a higher incidence of adverse events [69]. Uncontrolled RA activity is also known to lead to complications such as infection, respiratory disease, and renal dysfunction [46,70,71]; thus, the appropriate control of RA disease activity is also linked to the prevention and management of complications. Therefore, LORA requires a more multifaceted approach than YORA.

**Figure 2:**
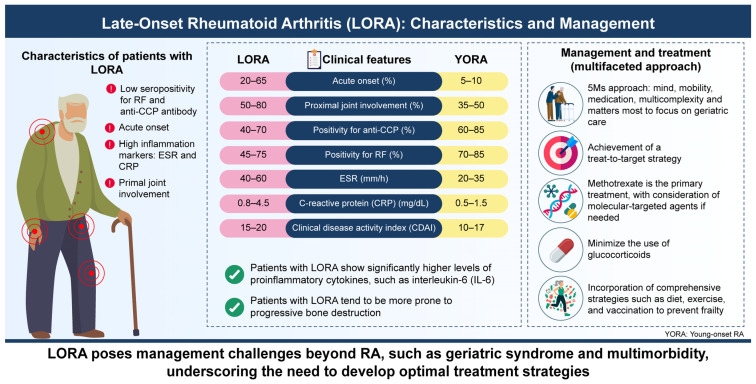
**Clinical characteristics and management of late-onset rheumatoid arthritis (LORA).** LORA tends to have low seropositivity, acute onset, high inflammation makers, and proximal joint involvement. LORA poses management challenges beyond RA, such as geriatric syndrome and multimorbidity, underscoring the need to develop optimal treatment strategies.

We summarize the clinical characteristics and management of LORA in Figure 2.

### 3.4. Future Prospectives for LORA

Although the number of patients with LORA is increasing rapidly with the aging society and is expected to increase further in the future, there are many unresolved issues relating to LORA. Remarkable advances have been made in the treatment of RA, but the evidence is mainly based on studies focused on patients with YORA and there is an urgent need to establish evidence for LORA. In the future, randomized controlled studies focusing on newly onset LORA and the accumulation of real-world data on LORA are needed to determine the optimal strategy. We have already initiated a registry study of LORA [72], which will provide information on the actual treatment situation and problems in LORA. In addition to clinical data, accompanying basic research, such as single-cell analysis using synovial specimens or focusing on immunosenescence in patients with RA, will help determine optimal treatment strategies by elucidating the pathological differences between LORA and YORA.

## Figures and Tables

**Table 1:** Molecular targeted therapy for rheumatoid arthritis.

TNF inhibitors	Infliximab
	Etanercept
	Adalimumab
	Golimumab
	Certolizumab pegol
	Ozoralizumab
T cell co-stimulation modulator	Abatacept
IL-6 receptor inhibitors	Tocilizumab
	Sarilumab
B cell depletion	Rituximab
JAK inhibitors	Tofacitinib
	Baricitinib
	Peficitinib
	Upadacitinib
	Filgotinib

IL-6: interleukin-6; JAK: Janus kinase; TNF: tumor necrosis factor.

**Table 2:** EULAR definition of difficult-to-treat rheumatoid arthritis (D2T RA).

1	Treatment according to European League Against Rheumatism recommendation and failure of ≥2 b/tsDMARDs (with different mechanisms of action) * after failing csDMARD therapy (unless contraindicated).†
2	Signs suggestive of active/progressive disease, defined as ≥1 of:
	a. At least moderate disease activity (according to validated composite measures including joint counts; for example, DAS28-ESR > 3.2 or CDAI > 10).
	b. Signs (including acute phase reactants and imaging) and/or symptoms suggestive of active disease (joint related or other).
	c. Inability to taper glucocorticoid treatment (below 7.5 mg/day prednisone or equivalent).
	d. Rapid radiographic progression (with or without signs of active disease). ‡
	e. Well-controlled disease according to above standards, but still having RA symptoms that are causing a reduction in quality of life.
3	The management of signs and/or symptoms is perceived as problematic by the rheumatologist and/or the patient.

All three criteria need to be present in D2T RA. * Unless restricted by access to treatment due to socioeconomic factors. † If csDMARD treatment is contraindicated, a failure of ≥2 b/tsDMARDs with different mechanisms of action is sufficient. ‡ Rapid radiographic progression: change in van der Heijde-modified Sharp score ≥5 points at 1 year. Abbreviations—b: biological; CDAI: clinical disease activity index; cs: conventional synthetic; DAS28-ESR: disease activity score assessing 28 joints using erythrocyte sedimentation rate; DMARD: disease-modifying antirheumatic drug; mg: milligram; RA: rheumatoid arthritis; ts: targeted synthetic.

**Table 3:** Prevalence and clinical characteristics of D2T RA.

Reference	Prevalence % (n)	Relevant Factors
Roodenrijs NMT [15]	N/A	Low socioeconomic status Young onset Multiple comorbidities Fibromyalgia Depression/anxiety Non-adherence Alcohol use Limited drug option
Takanashi S [16]	10.1 (173/1709)	Female Long disease duration Delayed bDMARDs/JAKis initiation Low body weight RF positivity Anti-CCP positivity Contraindication or intolerance to MTX Admission history of infection Multiple comorbidities Chronic kidney disease Lung disease
Watanabe R [17]	7.9 (53/672)	High RF titer (≥156.4 IU/mL) High baseline disease activity Lung disease
Messelink MA [18]	6.6 (123/1873)	N/A
Ochi S [19]	16.6 (353/2128)	N/A
Yoshii I [20]	21.5 (71/330)	Young onset Long disease duration High RF titer High anti-CCP titer High RDCI High baseline disease activity Worse QOL score Worse radiological progression
Giollo A [21]	24.9 (48/193)	Delayed MTX treatment Long-term glucocorticoid use
Novella-Navarro M [22]	13.7 (122/893)	Young onset
Leon L [23]	5.9 (35/631)	Young onset RF positivity High baseline DAS28 High baseline HAQ Dyslipidemia Liver disease DMARD monotherapy
Hecquet S [24]	23.8 (76/320)	Low socioeconomic status Long disease duration RF positivity Diabetes mellitus Interstitial lung disease Non-MTX use
Garcia-Salinas R [25]	27.5 (76/276)	High RF titer Anti-CCP positivity/ high titer Bad baseline HAQ Presence of erosion
Jung JY [26]	11.7 (271/2321)	Young age Long disease duration Low RF Non-csDMARDs use Rheumatoid nodule Cardiovascular disease Worse RAPID3
David P [27]	16.8 (247/1469)	N/A
Michitsuji T [28]	14.0 (43/307)	N/A
Bertsias A [29]	19.9 (251/12664)	Female Young age at bDMARDs/JAKis initiation RF negativity Shorter disease duration Osteoarthritis DAS28 at bDMARDs/JAKis initiation
Alp G [30]	8.9 (27/302)	Young onset Long disease duration Sjögren syndrome Extra-articular manifestation Comorbidity Fibromyalgia High RF
Watanabe R [31]	12.4 (450/3623)	Young onset High RF Comorbidity High disease activity Diabetes mellitus

Anti-CCP: ant-cyclic citrullinated peptide antibody; bDMARDs: biological disease-modifying anti-rheumatic drugs; csDMARDs: conventional synthetic disease-modifying anti-rheumatic drugs; DAS: disease activity score; D2T RA: difficult-to-treat rheumatoid arthritis; HAQ: health assessment questionnaire; JAKi: Janus kinase inhibitor; MTX: methotrexate; N/A; not available; RAPID3: Routine Assessment of Patient Index Data 3; QOL: quality of life; RDCI: rheumatic disease comorbidity index; RF: rheumatoid factor.

**Table 4:** Subgroup of D2T RA.

Reference	Subgroup and Characteristics
Roodenrijs NMT [15]	Non-adherence and dissatisfaction - Treatment non-adherence - Depression
	Pain syndrome and obesity - Smoking - Obesity - Fibromyalgia - Anxiety - High tender joint count
	True refractory RA - Erosion - Rheumatologist’s with to intensify treatment (where no further treatment options exist)
Takanashi S [16]	Multidrug resistance - Failure to 3 ≥ bDMARDs/JAKis - High tender joint count - High physician global assessment
	Comorbidity - Older age - History of admission due to infection - High RDCI - Long disease duration - Small physique
	Socioeconomic - Difficulty in initiation of bDMARDs/JAKi due to economic reason
David P [27]	Persistent inflammatory refractory RA (PIRRA) - High swollen joint count - High CRP - Worse DAS28-CRP
	Non-inflammatory refractory RA (NIRRA) - High BMI and more obese people - High fibromyalgia prevalence

BMI: body mass index; bDMARDs: biologic disease-modifying anti-rheumatic drugs; CRP: C-reactive protein; DAS: disease activity score; JAKi: Janus kinase inhibitor; RA: rheumatoid arthritis; RDCI: rheumatic disease comorbidity index.

**Table 5:** Clinical characteristics of late-onset rheumatoid arthritis.

	Young-Onset RA	Late-Onset RA
Sex (female), (%)	70–85	50–80
Acute onset, (%)	5–10	20–65
Proximal joint involvement, (%)	35–50	50–80
Positivity for anti-CCP, (%)	60–85	40–70
Positivity for RF, (%)	70–85	45–75
TJC	2–6	5–6
SJC	2–4	4–8
PGA, mm	35–50	35–65
EGA, mm	30–60	30–60
ESR, mm/h	20–35	40–60
CRP, mg/dL	0.5–1.5	0.8–4.5
CDAI	10–17	15–20
DAS28-ESR	4–5.5	5–6
HAQ-DI	0.4–0.7	0.7–1.5

Anti-CCP: anti-cyclic citrullinated peptide; CDAI: clinical disease activity index; CRP: C-reactive protein; DAS: disease activity score; EGA: evaluator global assessment; ESR: erythrocyte sedimentation rate; HAQ: health assessment questionnaire—disability index; RF: rheumatoid factor; SJC: swollen joint count; TJC: tender joint count; PGA: patient global assessment.
